# Understanding effective approaches to addressing the common challenges faced by global health networks: Mobilising multi-stakeholder networks to address the upstream determinants of maternal health in five low- and middle-income countries

**DOI:** 10.7189/jogh.13.04044

**Published:** 2023-06-09

**Authors:** Jewel Gausman, Ana Langer, R Rima Jolivet

**Affiliations:** Department of Global Health & Population, Harvard T.H. Chan School of Public Health, Boston, Massachusetts, USA

## Abstract

**Background:**

Past case studies on global initiatives to address maternal health and survival have focused on global health networks, identifying four essential tasks that define their ability to successfully enact change. We applied the conceptual framework of global health networks at the country level to organisations sharing concerns on how to address national maternal health and the upstream determinants of maternal survival in five countries and explored how they addressed these four essential tasks.

**Methods:**

We conducted focus group discussions and key informant interviews with 20 members of national maternal health multi-stakeholder networks in Bangladesh, India, Mexico, Nigeria, and Pakistan. We drew on the principles and essential components of appreciative inquiry, an assets-based action research methodology that emerged from positivist theories of organisational development to understand how the networks addressed the four tasks. We used a deductive content analysis approach, developing initial themes based on pre-designed codes corresponding to the four tasks faced by global health networks and later identifying emergent themes in the four areas of the framework.

**Results:**

We identified themes related to each of the four tasks. Participants emphasised the need for structure and focus in defining the problem, strengths associated with network diversity, and the network’s ability to pivot and redefine the problem to align with other sweeping priorities, such as COVID-19 pandemic. Themes related to inspiring action centred on aligning the issue with ongoing local and global initiatives, cultivating a sense of group ownership, and defining success incrementally. Themes related to forging alliances emphasised needing to engage high-level leadership, being opportunistic about timing, reducing barriers to participation by external players, and identifying rewards for participants. Themes related to establishing a governance structure centred on needing strong structure and organisation, cultivating individual commitment, sustaining advocacy efforts, and obtaining funding.

**Conclusions:**

Our results demonstrate that challenges commonly faced by global health networks are also relevant to networks operating on a national scale and may offer them strategies for future national networks to consider adopting to address these challenges.

Sustainable Development Goal (SDG) 3 focuses on ensuring healthy lives for all [1]. A crucial step to achieving it is ending preventable maternal mortality is critical, which is why target 3.1 is reducing the global maternal mortality ratio to less than 70 per 100 000 live births by the year 2030.

In 2015, the World Health Organization (WHO) released a report entitled “Strategies toward ending preventable maternal mortality” (EPMM Strategies), which outlined global targets and strategies for reducing maternal mortality in the 2015-2030 SDGs era [2], with a special focus on human rights and system performance to eliminate disparities in access, quality, and outcomes of maternal care both within and between countries. The strategies highlight 11 EPMM key themes grounded in fundamental human rights principles of equity, non-discrimination, transparency, participation, and accountability. They represent the full, broad spectrum of determinants of maternal health and survival, including social/structural, political, economic, and health system-level determinants. A comprehensive monitoring framework tied to each of these themes was developed to track national and global progress in improving maternal health [3,4].

In September 2017, the Women & Health Initiative of the Harvard T. H. Chan School of Public Health launched the Improving Maternal Health Measurement Capacity and Use (IMHM) project aimed at helping countries and global development partners plan, track and accelerate progress towards EPMM by further strengthening the framework’s indicators and their use through a variety of activities. Through the project, seven consultations (known as National Dialogues) were conducted to better understand national priority areas for improvement related to maternal health and to support the adoption and use of EPMM indicators in national-level monitoring frameworks to drive improvement in those self-identified priority areas. Each National Dialogue included approximately 40-50 stakeholders representing expertise for and commitment to each of the 11 EPMM key themes, including representatives from the Ministry of Health, UN and donor agencies, development partners, civil society advocates, and others from outside the health sector. The National Dialogues took place in seven countries: Bangladesh (February 2019), Cote d’Ivoire (November 2018), India (April 2019), Kenya (July 2018), Mexico (July 2019), Nigeria (March 2020), and Pakistan (October 2019).

The National Dialogue organisers hoped to use the platform as a catalyst to create a lasting multi-stakeholder network that would continue to drive the achievement of the established priorities and commitments beyond the one-day event. The Dialogues were organised by planning committees consisting of global and country-level project staff, representatives from each country’s Ministry of Health, representatives from UN Agencies and local government entities from relevant sectors outside health, as well as other various active country stakeholders who were selected to reflect the range of issued highlighted in the EPMM 11 key themes; all participants were national actors dedicated to advancing maternal and child health priorities. The planning committees were central to bringing stakeholders from participating countries together to engage them in the National Dialogues, strengthening commitments and supporting the shared agenda. While the agenda in each National Dialogue was country-specific, the desire to increase the attention paid to the upstream determinants of maternal health was part of a larger, global initiative.

Global initiatives to address maternal health and survival have been the subject of past case studies focused on understanding global health networks [5,6]. Global health networks are defined as “cross-national webs of individuals and organisations linked by a shared concern to address a particular health problem global in scope” [7]. They simultaneously serve policy, knowledge creation, and advocacy functions to bring about improvement in their domain of focus [8]. Past research has explored how global health networks seeking to address issues related to safe motherhood have encountered difficulty in gaining traction, partially because of issues relating to the severity of the problem on a population level, a lack of widely accepted approaches to measurement and monitoring, and limited consensus on intervention prioritisation [5]. Four essential tasks have been identified that define the ability of global health networks to successfully enact change: generating consensus on defining the problem and how it should be addressed, positioning the issue in a way that inspires action, forging alliances with players within and outside of the health sector, and establishing a persistent organisational structure to facilitate collective action on the issue [9].

We aimed to apply the conceptual framework of global health networks by mapping their four essential tasks to national webs of individuals and organisations sharing concerns on how to address national maternal health and survival so as to understand its relevance to national-level efforts. By focusing on how the National Dialogue planning committees view the effort to foster and sustain effective health networks in each of their contexts, we compare experiences and lessons learned across countries and explore whether and how they have been able to achieve the four essential tasks facing global health networks, to rally stakeholders over time in support of a common objective: the integration of EPMM themes and indicators into national level strategic plans, policies, programming, and/or monitoring frameworks.

## METHODS

### Methodological approach

Our methodology is grounded in the principles and essential components of appreciative inquiry (AI), an assets-based action research methodology that emerged in the 1980s out of positivist theories of organisational development [10]. Traditional deficit-based methodologies of organisational development focus on “the root causes of failure” [11], viewing organisations as broken entities that are defined by their weaknesses and problems, leading critics to argue that such approaches may become self-fulfilling and hamper future progress [12]. In contrast, AI encourages a deeper understanding of the “root causes of success” [11] to construct a future reality that embraces positive elements of change. It is based on the idea that understanding organisational challenges by focusing on achievements, peak experiences, and best practices is a more straightforward approach to identifying ways that organisations can improve than traditional approaches that are characterised by a preoccupation with problems and weaknesses [13]. By engaging stakeholders in transformative dialogue, AI values local knowledge and indigenous solutions first to create a belief that organisations have the power to change and then to empower actors to create that change [14].

While outlining a wide variety of approaches, most current literature includes four essential components of AI, which underlie what is known as the “AI 4-D process.” The four “Ds” are discovery (appreciating and valuing the best of what is), dream (envisioning what might be), design (dialoguing what should be), and destiny (innovating what will be) [15]. The implementation of these four components is flexible and has been widely adapted to meet the specific needs of various settings and participants [16]. For each of the four “Ds”, AI emphasises the use of narrative discussion and storytelling through which participants can develop a rich understanding of past experiences and visions for the future [17,18].

AI has been used extensively as an approach to programme evaluation across disciplines, including in applications related to health service delivery and system performance involving diverse stakeholder groups [16]. One such example is the Nepal Safer Motherhood Project, designed to change attitudes and improve accountability of health workers; the project used AI to harness group cooperation and to encourage new ideas and learning [14,19]. Elsewhere, AI was used to engage a broad group of actors during a multi-stakeholder conference focused on eliminating racial disparities in birth outcomes and to set a future agenda for action [20].

We used the principles of AI to understand how the IMHM National Dialogue planning committees addressed the four essential tasks that must be realised by successful global health networks. By using a positivist rather than a deficit-based approach, we aimed to capture achievements, positive experiences, and best practices with the goal of using these data to fuel continued progress. Rather than framing the four essential tasks of global health networks as challenges or limitations that inevitably impede progress, we used AI to engage the planning committees working to foster and sustain global health networks in their settings in a strength-finding reflection on their assets and positive experiences to date and what future progress may look like. We expected this process to generate important lessons to strengthen the current health networks and inform future programming in countries where the National Dialogues occurred and in those that may wish to undertake similar work in the future.

### Data collection

We used mini focus group discussions (FGDs) consisting of three participants per setting [21] and key informant interviews (KIIs) to maximise stakeholder participation. All FGDs were conducted in English, except for those in Mexico, which were conducted in either Spanish or English, depending on the fluency of the participants. We collected data between March and June 2021. Due to the limitations of the COVID-19 pandemic, all FGDs and KIIs were held remotely using Zoom. The facilitator followed a semi-structured discussion guide based on the four “Ds” to enable participants to reflect on their experiences. The facilitator had been involved at the global level in supporting the organisation and implementation of the National Dialogues and was known by the participants. Participants were informed that we were interested in conducting a strength-finding exercise from a positivist perspective, rather than focusing on deficits.

Each FGD was organised in a way that aligned with the AI 4-D process and was cantered on the four essential tasks of global health networks. For example, during the discovery phase, participants were asked about their experience defining the problem of maternal mortality in their country and how they thought the National Dialogues were effective in addressing it. As part of the dream phase, participants were asked to reflect on how the National Dialogues inspired action towards priority issues related to maternal mortality in their country and what ideas that have fostered continuing progress towards accomplishing country-level goals. The discussions during the design phase focused on how the National Dialogues forged connections within and outside the health sector, what experiences were most successful, and how existing networks could be expanded to create a stronger alliance. Last, the destiny phase focused on any past and ongoing efforts to establish a formal institutional body or informal network to drive progress in achieving the commitments made in the National Dialogues, while generating ideas for how to strengthen collective action and accountability. KIIs took approximately one hour to complete and FGDs took approximately one and a half hours.

### Study participants

Study participants were members of the planning committees in countries where the IMHM National Dialogues took place. Planning committees ranged in composition, but typically included members of the government, academia, non-governmental organisations, United Nations agency counterparts, and maternal health advocates. We purposefully selected the participants to include a range of perspectives. Due to the COVID-19 pandemic, we only included five of the seven countries in which the National Dialogues were held (Bangladesh, India, Mexico, Nigeria, and Pakistan). All members of the planning committees were invited to participate in either a KII or FGD, depending on their availability. We aimed to include at least two participants from each country, but included anyone from the planning committees who expressed interest. Participants were invited by email, and we followed up once in the event of non-response. **Table 1** provides the number of participants from each of the five countries.

**Table 1:** Number of participants in each country in focus group discussions and key informant interviews

Country	Number of participants	Mode of data collection
Bangladesh	4	1 FGD, 1 KII
India	7	2 FGDs, 1 KII
Mexico	2	2 KIIs
Nigeria	3	1 FGD
Pakistan	4	1 FGD, 1 KII

FGD – focus group discussion, KII – key informant interview

### Data management and analysis

We audio/video recorded each FGD and asked the participants to use video when internet connectivity allowed. The content of each FGD was transcribed and subsequently verified by another member of the research team for quality assurance.

We used a deductive content analysis approach in the data analysis, developing initial themes based on pre-defined codes, designing them to correspond to the four tasks faced by global health networks. Pre-designed codes were as follows: “Essential Task 1: Generating consensus on defining the problem and how it should be addressed”, “Essential Task 2: Positioning the issue in a way that inspires action”, “Essential Task 3: Forging alliances with players within and outside of the health sector”, and “Essential Task 4: Establishing some sort of governance or institutional body to facilitate collective action on the issue”.

Following the initial coding using pre-designed codes, the data were coded a second time for each of the four challenges identified a priori to identify emergent themes in the four framework areas [22]. We conducted data analysis using Dedoose [23].

Our research and reporting the Standards for Reporting Qualitative Research (SRQR) [24].

### Ethical approval

We obtained ethical approval from the Institutional Review Board at the Harvard TH Chan School of Public Health. All participants provided verbal informed consent to participate.

## RESULTS

### Essential task 1: Generating consensus on defining the problem and how it should be addressed

#### Theme 1.1: Need for focus and structure

Participants described that starting with a clear focus and applying a structure for deliberations helped them better understand the problem and prioritise next steps for action. Several participants thought that using an evidence-based framework (i.e. the EPMM key themes and indicators) gave participants a common understanding on which to begin building consensus on defining the problem of maternal mortality in their own country.

In terms of the [EPMM] framework, these are the key things that need to be discussed, and certainly they've already been researched, so I think that that was a helpful framework for us to think through [the issue]. *– Key informant, Bangladesh*

Others emphasised that having a shared background framework was especially important given that there was such a wide range of stakeholders present.

…that overall understanding was very helpful which put everybody in the same platform, because there were people from different backgrounds. *– Key informant, India*

Participants thought that focus and structure were equally important in developing an action plan or a “roadmap.” Participants reflected on how building consensus within the group was eased by having:

…a really focused agenda with some clear short and midterm goal posts and accomplishments. *– Key informant, Mexico*

In Nigeria, participants reflected on how having a clear plan for action supported their advocacy efforts after the meeting to get high-level support:

After this meeting, we went back to the [Federal Ministry of Health]... and presented our key feedback from this meeting...he was supportive... *– Key informant, Nigeria*

Structuring the action plan in a way that it reflected different stakeholders’ strengths was thought to be particularly effective in coordinating efforts within the network.

[W]e were very able to clearly kind of map that and try to support and help each other so that we can complement each other’s activities, and support the government in achieving their overall goals globally, to which they have committed. *– Key informant, Pakistan*

Further, it was thought that drawing on each other’s strengths to build on repeated efforts by multiple stakeholders was a benefit of having a cohesive, long-term action plan, recognising that part of the goal is to have conversations, especially with government, that can evolve over time as the networks’ activities progress.

Especially the ministry looks at the evidence and make decisions on the basis of what what's a priority, then it is important...to sit with them..[to] plan some of these analytical pieces and ask the right questions in that format and then come up with the solutions that could fit...If we are actually looking for how the network or multiple stakeholders can actually come together to mobilize the conversations, which may influence the thinking and the government, then that's a kind of another route that one can take... and that will be a little longer, and the conversations can still happen. *– Key informant, India*

#### Theme 1.2: Diversity within the network helped to reshape the problem

Broad representation from multiple stakeholders with different backgrounds and expertise was thought to enrich how the group defined the problem. Participants with different backgrounds highlighted new dimensions that may not have otherwise been discussed or addressed by the group. Diversity within the group helped to *expand the space* to engage more players in a meaningful way, as described by a participant in Mexico. In Nigeria, a participant emphasised that having a clear understanding of the issue structured on a framework and built upon a diverse group of stakeholder perspectives made this attempt at cross-sectoral collaboration more successful than past ones due to its creation of a cohesive group with a deep understanding of the problem.

Planning the EPMM dialogue, emphasized to us the importance of multi-sectoral approach to addressing maternal health, especially in Nigeria. So [in the past], we've had cases where different sectors are doing different things, and there is no coherent result. During the planning, we saw the importance of bringing in all the sectors, the Ministries of Data Research and Planning, the Ministries of Health, the Ministries of Education, those in finance, and the Bureau of Statistics themselves, and they actively participated in that meeting. Now, what that has done for us is that since the meeting we've had a closer working relationship with the Department of Family Health, in the Ministry of Health...and most of indicators that came up in our advocacy plan in that meeting, have often resonated, and the discussions [have been] moving forward. *– Key informant, Nigeria*

A diverse network was described as key to fully understanding the problem from multiple angles in a way that supported complex action.

[I]f you want to carry a woman from one place to another, you need to have good roads, right? And so these are the things which are very, very key so you know a lot of these stakeholders need to be there to understand [the issue]... because there are areas where your network does not work. *– Key informant, India*

Similarly, involving diverse stakeholders from across sectors was viewed as necessary to bring new ideas into the group to tackle problems. For example, a participant in Nigeria explained, once the issue is established as a priority across the diverse network, achievements can be magnified:

So it just shows the wonders of having this kind of dialogues and partnering with different stakeholders, especially people that have this as a priority in their programming. And because they can actually take ideas – people actually come in to look for ideas ...and decide to push these in their different programs. So, for me that was one key learning actually having to work with the stakeholders to see how this is playing out. *– Key informant, Nigeria*

Last, having participants in the network from different levels, not only different sectors, also helped enrich the group’s understanding of the issue as it was being defined.

I remember in that workshop, the participants’ level was different, I mean there were participants from leadership-level, then managers-level, then mid-level, then also practitioners, so in that sense, there was a [deep] understanding of the issue. *– Key informant, Bangladesh*

#### Theme 1.3: The network’s ability to pivot and stay relevant in the face of new challenges

Participants from all countries emphasised that the network’s ability to pivot to new issues was fundamental. The COVID-19 pandemic was a pervasive example of how the multi-stakeholder networks saw the priorities identified at the National Dialogues reflected in a new and overwhelming challenge. A participant from India reflected that:

All those stakeholders reinitiated dialogue on some of the recommendations from the past to see what new challenges have posed, and how those challenges link to the recommendation from the National Dialogue.

Similarly, in Bangladesh, the network’s ability to identify new opportunities to come together in the face of COVID-19 reinforced the consensus generated from the meeting:

COVID is really a very challenging situation for Bangladesh, but it is also true that our history says that when our country has faced some challenge, we actually stood up boldly, so COVID, is actually giving that opportunity to strengthen our digital system... All issues that need to be connected with [priorities identified at the National Dialogue]. And more you can connect... all of us will be in common understanding and [develop] a common way to move. *– Key informant, Bangladesh*

Finally, in Nigeria, participants described that the COVID-19 pandemic made the priorities defined in the National Dialogue more urgent, and by pivoting to focus on the immediate concerns brought about by COVID-19, they were able to position their own agenda in a way that fostered action. A participant described that the members of the network hosted a listening session to better understand the issues faced by health workers and citizens, which ultimately were very closely aligned with the priorities that emerged from the National Dialogue:

So that dialogue, the feedback from that [listening session] formed a presentation that we took back to the Ministry of Health, telling them okay, this is what we have heard from the citizens and the health workforce. And it was accepted, the Department of Family Health, accepted that and we are hoping that they are going to act on that submission. *– Key informant, Nigeria*

### Essential task 2: Positioning the issue in a way that inspires action

#### Theme 2.1: Aligning with other local and global priorities and strategies

Finding ways to align the issues identified within national priorities and strategies was important for the networks to inspire action within themselves to make progress on their action plans. By first coming together under a shared problem statement with a joint action plan, the multi-stakeholder networks were able to make greater progress towards meeting their commitments jointly than they would have in acting alone, as it allowed them to leverage a larger programmatic platform while fostering joint ownership. Further, it caused the issue of maternal health measurement, a topical area that participants described as typically appealing to only a small group of technical stakeholders, to be institutionalised into regular discussions when complementary activities were being planned. A participant from Bangladesh described it as a domino effect, with one success leading to further ones across the network:

All the stakeholders, plus NGOs who are involved…could all be there, then once the indicators are selected, they could make it as part of their indicators of their projects. So, the government sees that yes, these projects are getting these indicators right, we can improve it within our system also. Because without some evidence, government won’t suddenly introduce something. *– Key informant, Bangladesh*

Finding a way to align the network’s goals with government priorities came up repeatedly as a way to inspire action. A participant from Nigeria explained that building on a shared agenda had become more important in recent years:

“Initially, we would say ‘Hey, government, this is what we plan to do!’ We're doing this!’ but now the question is, what strategic plan does this contribute to and if you can't find where this contributes, it’s actually hard for you to just come in you know, with your agenda and all of that to drive that.” *– Key informant, Nigeria*

Aligning the relevant issues at national level within the context of global priorities was also seen as a strategy to drive progress towards achieving the network’s agreed upon action plan. Several participants referenced the SDGs and how the cross-sectoral focus on them has helped garner attention to the network’s goals across a diverse network.

So, [the Sustainable Development Goals] SDGs are really kind of landmark thing in Bangladesh...Maternal health is not only a Ministry of Family Planning health issue; this is beyond that. It has to cover education, awareness, social safety net, where a poor mother can receive voucher, a maternal health voucher. So, our understanding that this is not a health focused ministry, it goes beyond. So, SDG is actually helping us in that. *– Key informant, Bangladesh*

#### Theme 2.2: Building on external opportunities to reinvigorate action

Participants discussed that taking advantage of opportunities or platforms from outside the network was one way in which they have successfully continued to keep the network focused on realising the actions set out in the meeting. For example, participants said that they leveraged meetings that engage certain members of the network to reinvigorate the discussion on the commitments agreed upon in the National Dialogues. For example, a participant from Nigeria described efforts to engage stakeholders at the regular meeting of a working group focused on a separate initiative:

[That working group] brings most of the stakeholders into one meeting. The meeting holds quarterly so we are also able to reenter it... So there's room for discussions and you know, I remember the last meeting, we're able to make reference that “this dialogue happened and this is what was said,”...So the good thing is that we have this network where these stakeholders are actually able to come together and I think somehow the [onus] is on also us to make sure we are able to retrace the learnings from the dialogue. *– Key informant, Nigeria*

#### Theme 2.2: Being at the forefront of a wave

Network members were more likely to feel motivated when they felt that the issues they were focused on belonged to a process or initiative larger than the network itself. Some participants described this as forging new connections between the network and individuals who were pushing out new information or research on the topics prioritised in the National Dialogue. A participant in Mexico linked this to:

…the excitement of being able to work where you feel that you're at the forefront of a wave. *– Key informant, Mexico*

Participants in Bangladesh thought that timing the National Dialogue with the increased high-level political attention being given to maternal health was important. In India, one participant gave an example of how the National Dialogue aligned with large scale efforts to establish a cadre of midwives, which enabled the network to engage with an expanded group of technical experts who were pushing that issue while continuing to reinforce the consensus that was built during the meeting.

#### Theme 2.3: Defining success in bits and pieces

Feeling successful was also thought to be important to keep the network motivated and focused; however, several participants acknowledged the challenges involved in defining success in a multi-sectoral group given that successes are not immediately visible and are the result of many different actors. Further, the network’s successes were not typically on a large scale, but rather occurred in a piecemeal fashion. Some participants described that the network would be reinvigorated when small successes were achieved, such as seeing certain priorities or indicators being integrated into government programmes; however, there was recognition that specific attribution was not possible. Maintaining motivation was integral to maintaining action. To achieve this, some participants suggested finding a way to provide feedback to the network when achievements occurred.

[W]hat I feel is that the process should be a continuous process where whatever the steps have been taken, or whatever the achievements have been gained, they should be displayed or disseminated at certain forum because people... they are involved in the initial process, and then what comes out of that or what progress is there, they are not informed, timely, and this causes lack of interest. *– Key informant, Pakistan*

### Essential task 3: Forging alliances with players within and outside of the health sector

#### Theme 3.1: Simplifying language to make network appealing to broader audience

One strategy echoed by participants across countires was the need to simplify the language used to facilitate understanding across sectors so as to increase broad participation in the network. They agreed that the language used within the health sector and (more narrowly) within groups of stakeholders focused only on the issue of maternal health tends to be overly technical, which ultimately excludes participants or causes those from other sectors to disengage.

Because the technical language we use is not the same language they use... so if you want to really talk about these themes and the indicators – cross cutting issues – we have to use very simple language. *– Key informant, Bangladesh*

#### Theme 3.2: Establishing linkages at the right time with high-level decision makers

Engaging high-level leaders, especially individuals with political power or access to financial resources, was thought to be essential to the network’s success.

I think a critical success factor was a clear link to decision-makers and people who had access to budget so that it felt like a policy advocacy group where discussions could translate into actionable change that had an impact on the field level. *– Key informant, Mexico*

However, simply engaging high-level decision makers was not enough; participants described that both strategy and timing were important factors to consider when bringing them into the network.

Regarding strategy, participants referenced the need to be prepared with evidence when approaching politicians and other high-level politicians in order to successfully engage them in the network.

You know, evidence takes time and they these [politicians] are in a hurry. They come with a plan that you know, ‘we will make changes in a particular area,’ like that, and they try to find out and once they do, they do not get that [research takes time], and then they think that, ‘Okay, there is [no evidence]...’But the problem is that you know, we as technical people can say that, but for politicians, if they have five years and they can't wait for the evidence to come up... *– Key informant, India*

Timing was also key when engaging high-level decision makers. Timing stakeholder engagement when similar issues were already on the agenda was thought to be an important part of the strategy, and helpful in focusing attention on the issues.

The timing of it was a clear direction politically of a consolidated national prioritization for maternal health, which brought a lot of stakeholders together... *– Key informant, India*

#### Theme 3.3: Bringing in new members over time to re-energise the group

Participants emphasised that the network should not be stagnant, but should have a strategy to bring in new members from across different sectors over time to re-energise the group’s thinking and inject new excitement.

One person leaves that institution, or a young person graduates and leaves that University, [if] the University is on board, they're going to give us another person, and we can involve them, we can institutionalize this process. *– Key informant, Pakistan*

Similarly, participants described the need to continually engage in outreach to new institutions and organisations to keep the network engaged in the field and relevant. Such a strategy was thought to be especially useful in expanding the network beyond even groups that were targeted by outreach.

Bring[ing] people [in] from different sectors...help us build new partnerships, which we have continued to engage since the [National] Dialogue. It has also made some organizations that don't even really know what we do, really understand...it also amplified what we do and who we are to a lot of other organizations. I remember somebody from the Ministry who we haven't engaged with before came and said, ‘Oh, I really like your organization, you guys seem really organized,’ and he had invited us for subsequent meetings after that... It has helped us build new partnerships; it has also kept us in that space. *– Key informant, Nigeria*

#### Theme 3.4: Need for catalytic/transactional exchange

A final way participants described for bringing in new members to the network was to ensure opportunities for members to get something tangible back for their participation. While generally membership in the network came from being passionate about the issue, it was also recognised that it had a transactional component.

One of the important areas is the question that people might ask, especially in the private sector, NGOs, and the media, is ‘What is in it for them?’ This is a sort of a question that we normally face, that why should they spend time with public sector people if there's nothing for them? *– Key informant, Bangladesh*

Another participant in Mexico described that clear rewards for participation in networks was an important incentive for members to join and stay engaged:

And then I saw that engaging in that group was like a ticket to something else, maybe it was ticket to go to “Women Deliver.” Maybe it was a ticket to get X, you know, but it was kind of like this idea of a catalytic exchange or a way or whatever, but there was a transactional thing there. *– Key informant, Mexico*

### Essential task 4: Establishing some sort of governance or institutional body to facilitate collective action on the issue

#### Theme 4.1: Building a platform on an existing initiative

Creating a clear group structure was thought to promote accountability within the network. Some participants described an ideal group structure to be more like a platform, and their descriptions of that structure varied from being relatively loosely defined to being more established. A common strategy to obtain a cohesive structure was to build it upon either an existing initiative or network, as described by a participant in Pakistan:

I think that there are a number of forums or networks already available, and all you need to do is to basically, get in touch with them and invite the key person responsible...and then, once they are part of the discussion...you basically form the network... *– Key informant, Pakistan*

As an example of a more defined platform, a participant in Mexico described a network structure built on a national programme:

[The network] links many institutions that are also law enforcing institutions, so it's a large multi-sectoral effort. And we're legally bound by a national program and by the...law and so we're all working towards that common agenda because we're legally bound to it and there is actually no funding to coordinate these efforts, but there is a structure for it, so even though there's no dedicated funding, there is a structure that allows for the system to work together. *– Key informant, Mexico*

#### Theme 4.2: Creating a sense of individual commitment to the group

Participants believed that ensuring that members felt committed to the network’s success was essential to facilitating collective action. Sharing successes was one way to do this; however, participants thought that finding ways to enable network members to feel ownership over the successes achieved was important. Establishing a mechanism for network members to share their individual contributions and discuss their specific role in the group’s achievements would help foster that sense of individual ownership that participants thought was necessary, while simultaneously helping develop a shared commitment to the network.

I was quite comfortable in my space and I used to give space to other people. That's some very major thing, let me tell you, because technical people are quite insecure, let me be honest. And if you don't give that space, people don't want to come to you. And when you allow and you give platform to these guys, who are doing a lot of good work... and actually take them and expose them to the limelight which, is due to them... a lot of good work is being done. *– Key informant, India*

Establishing an institutional body was also a way in which participants thought the network could better cultivate a sense of commitment to the group, as it would make the agenda more achievable. Some participants thought such an approach may be more easily realised by establishing a core team within the network so that roles and responsibilities are easily defined.

A sense of ownership or individual commitment to the group, a clear sense of roles and responsibilities, so a small enough group of people that there's a sense of role… that you're not being a participant, but there's this sense of agency. *– Key informant, Mexico*

#### Theme 4.3: Generating a driving force

Participants agreed that a leader or some force capable of pushing the group towards accomplishing its shared agenda was needed for the networks to be effective. Several different types of leadership structures were proposed, ranging from individual leaders to secretariats, but all believed that having some sort of formal direction was necessary to keep the network functioning and accountable. There was also a lot of emphasis on linking the network to established government initiatives to serve this function, in the absence of funding for a standalone leader. In Bangladesh, a participant described how such a leadership role could be played indirectly by an external government body:

Commitments from the government, who is the regulatory body... not in terms of, ‘We're going to do this’ [but] in terms of saying, ‘Yes, we’ll follow up. We'll have more meetings to see how it can be integrated, how [it] can be more focused. *– Key informant, Bangladesh*

However, a participant in Mexico described differences between how local networks function vs international networks on establishing an accountable leadership structure:

I’ve seen the groups that work internationally, where there is a secretariat or there's someone, that is moving the agenda forward, and [there is] usually someone [who has] been paid for that time. Then...locally, in terms of other networks that are national...[they] are usually linked to legal issues, so...there is something that legally binds you to responding in a network forum [to] some sort of common agenda. *– Key informant, Mexico*

#### Theme 4.4: Funding was a missing piece in establishing a governance structure

Nearly all participants reflected on the need for funding to support a network’s ongoing governance structure. Many participants said that the networks often relied on favours to move their agenda forward in the absence of funding, which was not sustainable. As described by a participant in Mexico:

It takes a lot of [effort to] coordinate these networks, even smaller networks, takes dedicated time and takes funding, and I think that's a critical ingredient. *– Key informant, Mexico*

When funding is available, then it works. And, most of the time, funding is not there...There is a leadership crisis ticking. *– Key informant, Bangladesh*

## DISCUSSION

Global health networks are critiqued for being often led by actors from the global north and having limited representation in their leadership by members from the South. Our results demonstrate, however, that the challenges commonly faced by global health networks are also relevant to networks operating on a national scale, which are all cultivated and led by local actors.

The internal framing of an issue within the network and its external positioning through how said issue is publicly portrayed are important factors related to network effectiveness [9]. It has been suggested that the maternal health community has been unable to frame the issue effectively across multi-stakeholder networks in order to gain traction; such challenges have been identified in relation to measuring progress, articulating precise strategies to address the problem, and understanding the issue in relation to the health and health systems concerns [5]. We found that, by centring the National Dialogues on the EPMM key themes and indicators and then leveraging the meeting to engage stakeholders in establishing consensus on a clear action plan, the networks in this study were able to overcome some of the challenges related to internal framing that have been faced by the global community. Having a common framework enabled the network to have an initial platform on which to build consensus in understanding the problem locally and agreeing on context-specific priorities for action, which was thought to be especially useful given the wide range of network actors present at each National Dialogue.

Our results also provide insight on how national networks to address maternal mortality have succeeded in positioning the issue externally to non-experts to gain support and motivate stakeholders to act. Despite efforts of global networks to position the issue of maternal mortality as a literal matter of life and death, they have often had limited success in gaining traction outside of the health sector [5]. Two important dimensions of external positioning emerged from our results. First, others within global governance frameworks have argued that policy coherence and inter-sectoral cooperation can come from the alignment of national priorities with global responsibilities [25]. By positioning the network’s goals within the SDGs, networks were able to effectively generate cross-sectoral interest. Second, past research has found that high-stakes issues, such as those associated with high mortality, morbidity, and social disruption, are likely to generate more robust networks [6]. Aligning the upstream determinants of maternal mortality within the context of the COVID-19 pandemic enabled the stakeholders to associate their issue with an issue that was of critical, global importance. In our study, participants described the catalysing effect that the COVID-19 pandemic had on strengthening their networks and gaining traction in achieving their agenda, which appears to be a policy window – a moment at which conditions aligned to present a strong opportunity to engage with leaders on an issue [5]. The fact that successful networks were able to pivot and re-frame the issue of maternal health to highlight its relevance within the pandemic response appears to have helped them to strengthen their standing.

Historically, global health networks seeking to address maternal mortality struggled to gain traction due to stove-piped initiatives that were perceived to be led by technical actors from the global north [5,6], as well as challenges involved in engaging a diverse network of stakeholders. Similar difficulties emerged in our research, but at a local level. Network members recognised that, to be effective in driving progress, their networks needed to reflect diverse expertise and interests, while also making outside actors feel welcome. Our research highlighted the strategies that the multi-stakeholder networks had to put in place to overcome the barriers they faced in diversifying their networks, such as by reducing the technicality of the language used.

Last, in terms of leadership, establishing the network’s legitimacy by linking its goals with ongoing government initiatives of platforms helped the network cultivate power to act and make progress towards its agenda. By comprising both state and non-state actors, it seemed that the networks in our study were able to more effectively harness the state power. While the subject of much debate in the literature on global health networks [26], our results point to an underlying need for buy-in from the government to strengthen the national networks described here.

Despite the networks’ ability to obtain government buy-in, ensuring the network itself had formalised leadership, either through individuals or a secretariat, was also seen as critical to its success and survival. While passionate individuals were needed to give the network a vision, there also needed to be a persistent driving force and abiding structure to ensure that network members were organised in a way that leveraged their strength to act efficiently and effectively.

This study has several strengths and limitations. It included diverse stakeholders from a wide range of countries facing different, context-dependant challenges related to maternal mortality. However, it was conducted during the COVID-19 pandemic, which made participation for some stakeholders difficult and likely reduced the number of participants. While we believe that our sample size is sufficiently large in each country to represent a wide range of voices – especially given the convergence of themes that were found across the study countries – our research could have benefitted from additional perspectives.

## CONCLUSIONS

Our study illustrates the applicability of the conceptual framework of global health networks at national levels, highlighting several effective approaches for national multi-stakeholder networks to address common challenges faced by global health networks. The themes we identified may be offer helpful strategies for future national networks in addressing these challenges.
